# iTRAQ-Based Quantitative Proteomic Comparison of Early- and Late-Passage Human Dermal Papilla Cell Secretome in Relation to Inducing Hair Follicle Regeneration

**DOI:** 10.1371/journal.pone.0167474

**Published:** 2016-12-01

**Authors:** Huan Zhang, Ning-Xia Zhu, Keng Huang, Bo-Zhi Cai, Yang Zeng, Yan-Ming Xu, Yang Liu, Yan-Ping Yuan, Chang-Min Lin

**Affiliations:** 1 Department of Histology and Embryology, Shantou University Medical College, Shantou, Guangdong, China; 2 Department of Cardiology, First Affiliated Hospital, Shantou University Medical College, Shantou, Guangdong, China; 3 Emergency Department, Second Affiliated Hospital, Shantou University Medical College, Shantou, Guangdong, China; 4 Tissue Engineering Laboratory, First Affiliated Hospital, Shantou University Medical College, Shantou, Guangdong, China; 5 Laboratory of Cancer Biology and Epigenetics, Department of Cell Biology and Genetics, Shantou University Medical College, Shantou, Guangdong, China; University of Ulm, GERMANY

## Abstract

Alopecia is an exceedingly prevalent problem that lacks effective therapy. Recently, research has focused on early-passage dermal papilla cells (DPCs), which have hair inducing activity both in vivo and in vitro. Our previous study indicated that factors secreted from early-passage DPCs contribute to hair follicle (HF) regeneration. To identify which factors are responsible for HF regeneration and why late-passage DPCs lose this potential, we collected 48-h-culture medium (CM) from both of passage 3 and 9 DPCs, and subcutaneously injected the DPC-CM into NU/NU mice. Passage 3 DPC-CM induced HF regeneration, based on the emergence of a white hair coat, but passage 9 DPC-CM did not. In order to identify the key factors responsible for hair induction, CM from passage 3 and 9 DPCs was analyzed by iTRAQ-based quantitative proteomic technology. We identified 1360 proteins, of which 213 proteins were differentially expressed between CM from early-passage vs. late-passage DPCs, including SDF1, MMP3, biglycan and LTBP1. Further analysis indicated that the differentially-expressed proteins regulated the Wnt, TGF-β and BMP signaling pathways, which directly and indirectly participate in HF morphogenesis and regeneration. Subsequently, we selected 19 proteins for further verification by multiple reaction monitoring (MRM) between the two types of CM. These results indicate DPC-secreted proteins play important roles in HF regeneration, with SDF1, MMP3, biglycan, and LTBP1 being potential key inductive factors secreted by dermal papilla cells in the regeneration of hair follicles.

## Introduction

Alopecia is an exceedingly prevalent problem that not only affects psychological well-being, but also endangers certain inherent functions of the skin [1]. However, currently available treatments, including drug therapies and human hair transplantation, have numerous limitations [2]. Recently, cell-based hair regeneration has been investigated as a possible alternative, and various experimental assays have shown that early-passage dermal papilla cells (DPCs) can induce hair follicle (HF) regeneration both in vitro and in vivo [2]. An HF is composed of epidermal (epithelial) and dermal (mesenchymal) compartments, and their interaction plays an important role in HF morphogenesis and growth [2, 3]. The dermal papilla (DP), formed at the base of the hair follicle, is a unique tissue surrounded by epithelial matrix cells and is essential in the control of hair growth, formation and cycling. The tendency to aggregate is one of the significant characteristics of DPCs and is associated with biological function. This ability to aggregate and capacity to induce HF differentiation is lost after several passages of culture [2]. The loss of the extracellular matrix (ECM) during culture could be responsible for the gradual loss of DPC inductive properties [1]. Keratinocyte conditioned medium and adipocyte lineage cell conditioned medium enables DPCs to aggregate and preserves their inductive activity [2, 4]. Although the molecular mechanisms of sustaining the inductive ability of DPCs have not been explored completely, the ability of conditioned medium to sustain the formation of aggregates implies that secreted proteins, from cells into the culture medium, may play roles in preserving the inductive ability of DPC.

To date, numerous studies have demonstrated that HF regeneration depends on a complex series of interactions mediated by DPCs through a paracrine mechanism [5]. DPC-derived factors have been reported to influence surrounding cells, which leads, in turn, to promotion of hair growth [6]. A previous *in vivo* study showed that CM from balding DPCs delays anagen onset in mice, but CM produced by normal scalp dermal papilla cells is able to stimulate growth of rat whisker cells [7]. Furthermore, DPCs encapsulated in alginate-polylysine-alginate (APA) still maintain the capacity for initiating the follicle regeneration [8, 9], implying that proteins secreted by aggregated DPCs play important roles in HF regeneration. Thus, the identification of paracrine factors in the DPC secretome would be of significant value for understanding the regulation of HF regeneration and identifying factors for therapeutic use.

HF morphogenesis depends on Wnt, Sonic Hedgehog (Shh), Notch, Bone morphogenetic protein (BMP) and other signaling pathways, and the interplay between epithelial and mesenchymal cells [3]. HF regeneration depends on activation of the Wnt and other underlying pathways, creating a microenvironment leading to BMP inhibition [3]. To better understand the influence of DPC-secreted cytokines on HF regeneration, we exploited recent advances in proteomics technology and bioinformatics to conduct temporal qualitative and quantitative secretome profiling of DPCs. Then we used stable isotope label-based isobaric tags for relative and absolute quantitation (iTRAQ) [10] to identify differentially-expressed secreted proteins between early- and late-passage DPC-CM. Moreover, we selected 19 differentially-expressed proteins, between the two types of CM, for further verification by multiple reaction monitoring (MRM) [11] to verify key candidate proteins for HF regeneration. We show that CM from aggregated DPCs, as well as early-passage DPCs, but not from non-aggregated DPCs or CM from late-passage DPCs, induces HF regeneration. We further identify the putative factors, responsible for HF regeneration, in DPC-CM. This work not only produces a database of secreted proteins from DPC, but will also help us identify novel factors that may be involved in regulating HF regeneration.

## Methods

### Culture of scalp cells

DPCs were isolated from human scalp tissue and subjected to primary culture and subculture [12]. Human scalp tissues were obtained with informed consent from the First Affiliated Hospital, Shantou University Medical College.The protocols used in the study were approved by the Hospital’s Biomedical Ethics Committee (permit number 2015–42), and all participants provided written informed consent. Third and ninth passage DPCs were used to represent early- and late-passage DPCs, respectively. Secreted proteins were prepared from harvested growth medium and subjected to iTRAQ.

### Preparation of secreted proteins from DPCs

Primary cultured DPCs were inoculated into 75 cm^**2**^ culture flasks containing DMEM (Invitrogen-Gibco) with 10% fetal bovine serum (FBS Invitrogen-Gibco) and incubated in a humidified 5% CO_**2**_ atmosphere at 37°C. After reaching confluence, cells were washed three times with PBS and incubated in serum-free DMEM for 48 h, after which the CM was harvested, centrifuged (3000 g, 4°C, 10 min) and filtered through a 0.22 μm filter (Millipore) to ensure removal of cells. The supernatant from each dish (10 ml) was transferred to an Amicon ultrafiltration unit (3 kDa MWCO, Millipore, Billerica, MA, USA) and concentrated to a final volume of 250 μl, and protease inhibitor cocktail was added at a ratio of 1:100 (v/v). The proteins were reduced with DTT (10 mM final concentration) at 56°C for 1 h, and then alkylated with iodoacetamide (55 mM final concentration) in the dark for 1 h. The reduced and alkylated protein mixtures were precipitated by adding 4× volume of chilled acetone and storage at -20°C overnight. After centrifugation at 4°C, 30000 g for 20 min, the pellet was dissolved in 0.5 M TEAB (Applied Biosystems, Milan, Italy) and sonicated on ice. After centrifuging at 30,000 g, 4°C for 20 min, an aliquot of the supernatant was taken for determination of protein concentration by using a bicinchoninic acid (BCA) protein assay kit (Pierce, Rockford).

### In vivo hair follicle induction by different passage DPC-conditioned culture medium

NU/NU mice (6 weeks old) were obtained from the Center for Research of Animals, Shantou University Medical College. All experiments were approved by the Ethics Committee on Research Animal Care at Shantou University Medical College (permit number SUMC2015-057). NU/NU mice (n = 16) were divided into 2 groups: (a) experimental group, injection of either passage 3 DPC-CM; (b) control group, injection of passage 9 DPC-CM. Mice were individually anesthetized with 1% sodium phenobarbital, and their abdomens were disinfected with iodine. Each mouse was subcutaneously injected with approximately 400 μl CM (2 μg protein/μl) into the dorsum. Mice were euthanized by CO_2_ inhalation after 5 days, and the implantation sites were biopsied for histology.

### Histology

Dorsal skins were harvested for histological analysis. Specimens were fixed with 4% paraformaldehyde, dehydrated through a graded series of ethanol, washed with xylene, and embedded in paraffin wax. Treated specimens were cut into 4 μm-thick sections and stained with hematoxylin and eosin for routine histology evaluation. Tissue was observed using bright field microscopy.

### iTRAQ analysis

#### iTRAQ labeling

A 100 μg aliquot of each sample solution was digested with Trypsin Gold (Promega, Madison, WI, USA), at a protein: trypsin ratio of 30:1 (w/w), at 37°C for 16 hours. After trypsin digestion, peptides were dried by vacuum centrifugation, then reconstituted in 0.5 M TEAB (pH 8.5) and processed according to the manufacturer’s protocol for 8-plex iTRAQ reagent (Applied Biosystems). Briefly, one unit of iTRAQ reagent was thawed and reconstituted in 24 μL isopropanol. Samples were labeled with the iTRAQ tags as follows: DP3-1,113; DP3-2,115; DP3-3,118; DP9-1,114; DP9-2,117; DP9-3,121. The peptides were labeled with the isobaric tags and incubated at room temperature for 2 h, and then labeled peptide mixtures were pooled and dried by vacuum centrifugation.

#### Strong cation exchange chromatography (SCX)

SCX chromatography was performed with an LC-20AB HPLC pump system (Shimadzu, Kyoto, Japan). The iTRAQ-labeled peptide mixtures were reconstituted with 4 mL buffer A (25 mM NaH_2_PO_4_ in 25% acetonitrile (ACN), pH2.7) and loaded onto a 4.6 X 250 mm Ultremex SCX column containing 5-μm particles (Phenomenex). The peptides were eluted at a flow rate of 1 mL/min with a gradient of buffer A for 10 min, 5–60% buffer B (25 mM NaH_2_PO_4_, 1 M KCl in 25% ACN, pH2.7) for 27 min,　and 60–100% buffer B for 1 min. The system was then maintained at 100% buffer B for 1 min before equilibrating with buffer A for 10 min prior to the next injection. Elution was monitored by measuring the absorbance at 214 nm, and fractions were collected every 1 min. The eluted peptides were pooled into 20 fractions, desalted with a Strata XC18 column (Phenomenex) and vacuum-dried.

#### LC-MS/MS analysis

Each fraction was resuspended in solvent A (5% ACN, 0.1% FA) and loaded onto a 2 cm C18 trap column in an LC-20AD NanoHPLC system (Shimadzu, Kyoto, Japan). Then, the peptides were eluted onto a 10 cm analytical C18 column (inner diameter 75 μm) packed in-house and separated with a 35 min main gradient starting from 2 to 35% B (95%ACN, 0.1% FA) at a total flow rate of 300 nL/min,

Data acquisition was performed with a Triple TOF 5600 System (AB SCIEX, Concord, ON) fitted with a Nanospray III source (AB SCIEX, Concord, ON) and a pulled quartz tip as the emitter (New Objectives, Woburn, MA). The MS was operated with an RP (Reverse Phase) of greater than or equal to 30,000 FWHM for TOF MS scans. For IDA (Information Dependent Acquisition), survey scans were acquired in 250 ms, and as many as 30 product ion scans were collected if exceeding a threshold of 120 counts per second (counts/s) and with a 2+ to 5+ charge-state. The Q2 transmission window was 100 Da for 100%. A sweeping collision energy setting of 35±5 eV, coupled with iTRAQ adjusted rolling collision energy, was applied to all precursor ions for collision-induced dissociation. Dynamic exclusion was set for 1/2 of peak width (15 s), and then the precursor was refreshed off the exclusion list.

#### Data analysis

Raw data files acquired from the TripleTOF5600 were converted into MGF files using Proteome. Discoverer 1.2 (PD 1.2, Thermo), [5600 msconverter], and the MGF file was searched. Protein identification was performed by using the Mascot search engine (Matrix Science, London, UK; version 2.3.02) against a human database containing 127497 sequences was downloaded from Uniprot database on June 17, 2015. For protein identification, a mass tolerance of 0.05 Da was permitted for intact peptide masses, and 0.1 Da for fragmented ions, with allowance for one missed cleavage in the trypsin digests. Gln->pyro-Glu (N-term Q), oxidation (M), and deamidation (NQ) were the potential variable modifications, and carbamidomethyl (C), iTRAQ8plex (N-term), iTRAQ8plex (K) were the fixed modifications. The charge states of peptides were set to +2 and +3. The automatic decoy database search was performed in Mascot by choosing the decoy checkbox in which a random sequence of the database is generated. To reduce the probability of false peptide identification, only peptides at a 95% confidence interval (P < 0.05) with a false discovery rate (FDR) estimation ≤1.04% were counted as being successfully identified. Each confident protein identification involved at least one unique peptide. For protein quantitation, it was required that a protein contains at least two unique spectra. According to the differences of these spectra, we performed a t-test to determine the significance of the differences of each protein between the different samples. The quantitative protein ratios were weighted and normalized by the median ratio in Mascot. The ratio between passage 3 DPC-CM and passage 9 DPC-CM was obtained directly based on the protein abundance for any given protein. Proteins were determined as differentially expressed if: i) only the ratios with *P*-value <0.05 were used, ii) the 3 repetitions were consistent, iii) at least 2 repetitions met a fold change of > 1.2.The analysis was performed by the Beijing Genomics Institute (BGI) (Shenzhen, China).

### Determination of secreted proteins

Previous studies employed bioinformatic tools to identify secreted proteins [13]. First, the primary sequence information of the identified proteins was used to submit to the web-based bioinformatics tools SignalP [14] and SecretomeP [15] to determine the probability of being a secreted protein; only those proteins with secretion probability above the default threshold values determined by the tools were used. Second, AmiGO, a web-based tool with an E-value threshold of 1E-5, was used to recognize proteins as extracellular by searching and browsing the GO database [16]. Third, protein matching was conducted by using the ExoCarta database that records proteins identified as being secreted products based on exosome data from previous studies [17].

### Multiple reaction monitoring (MRM) analysis

#### Peptide selection

In this project, we aimed to develop an MRM method for 19 target proteins. We used Skyline software [18] to select peptides of the target proteins, with an MS/MS spectral library (cut-off score: 0.95), which was generated on a TripleTOF5600 (AB SCIEX) search using Mascot (Matrix Science, UK) against a human database containing (127497 entries) was downloaded from Uniprot database on June 17, 2015.The Skyline file have been submitted to the Peptide Atlas SRM Experiment Library (PASSEL, http://www.peptideatlas.org/PASS/PASS00884).The 19 proteins had an MS/MS spectrum and unique peptides. Then we used a QTRAP 5500 (AB SCIEX) to verify whether the peptide had a co-elution chromatogram and the correct retention time. Finally, we developed an MRM method for the 19 proteins. Details of all the peptides used in this study and their corresponding proteins are available in S1 Table.

#### Setting the MRM transition

A spectral library of MS/MS data was generated on a TripleTOF5600 (AB SCIEX, Foster City, CA) and searched using Mascot v2.3 (Matrix Science, UK) against a human database that was downloaded from the Uniprot database and consisted of 127497 sequences. All 19 proteins had MS/MS spectra and unique peptides. Then we used a QTRAP 5500 (AB SCIEX) to verify whether the peptide had a co-elution chromatogram and correct retention time. Finally, we developed an MRM method for the 19 proteins. The data file was imported into Skyline software, where a library was built. The peptides were selected for MRM method development according to the following criteria: (1) the peptides had a unique sequence in the database, (2) a maximum m/z of peptide <1250 (limination of quadrupole scan), with a peptide length range of 5–40 aa, (3) the peptides lacked methionine, (4) had a carbamidomethyl group attached to residues and was without variable modification in the peptides, and (5) no missed cleavage by trypsin. We initially monitored 6 transitions per peptide to ensure specificity with the criteria that >5 y-ions had the same elution profile and were in the same ratios as the spectral library. The predicted retention time of targeted peptides was observed using an iRT strategy.

#### LC-MRM-MS

Samples were digested as described and spiked with 20 fmol of β-galactosidase for data normalization. MRM analyses were performed on a QTRAP5500 mass spectrometer (AB SCIEX, Foster City, CA) equipped with a Waters Nano Acquity Ultra Performance LC system. The mobile phase consisted of solvent A (0.1% aqueous formic acid) and solvent B (98% acetonitrile with 0.1% formic acid). Peptides were separated on a BEH130 C18 column (0.075 x 200 mm column, 1.7 μm; Waters) at 300 nL/min, and eluted with a gradient of 2%-40% solvent B for 30 min, and 40%-60% solvent B for 3 min, followed by a 2 min linear gradient to 80% solvent B and maintenance at 80% for 5 min. For the QTRAP5500 mass spectrometer, a spray voltage of 2100 V, nebulizer gas at 20 p.s.i., and a dwell time of 10 ms were used. Multiple MRM transitions were monitored using unit resolution in both Q1and Q3 quadrupoles to maximize specificity.

#### MRM method validation

The chromatograms of all transitions generated on QTRAP5500 were input into Skyline. The MRM method of a given protein was considered successfully developed only if the protein had at least one unique peptide that (1) was identified in the MS/MS spectral library (cut-off score >0.95), (2) had >5 fragment ions with the same elution profile and in the same ratios as the spectral library, and (3) had an accurate retention.

#### Data analysis

We used Skyline software to integrate the raw file generated by QTRAP 5500 (AB SCIEX). We used an iRT strategy to define the chromatography of a given peptide against a spectral library. The top three abundant transitions for each peptide was used for quantitation unless interference from the matrix was observed. Samples were spiked with β-galactosidase for label-free data normalization. We used MSstats with a linear mixed-effects model. *P*-values were adjusted to control the FDR at a cutoff of 0.05. All proteins with a *P*-value below 0.05 and a fold change larger than 1.5 were considered significant.

### Immunohistochemistry

For deparaffinization and rehydration, tissue sections were sequentially treated with xylene (5 min x 3), 100% ethanol (2 min), 95% ethanol (2 min), 85% ethanol (2 min), 75% ethanol (5 min) and distilled water (2 min). An Immunohistochemistry Accessory Kit (GK500705, Gene Tech, Shanghai, China) was applied for antigen epitope retrieval, secondary antibody incubation, following the manufacture’s recommendations, and color development. After blocking, the section was incubated overnight at 4°C with the primary antibody, anti-SDF1 (1:150, BA1389, Boster, Wuhan, China), anti-MMP3 (1:300, sc-6839,Santa Cruz Biotechnology, Santa Cruz, CA,USA), anti-biglycan (1:150, sc-27936,Santa Cruz Biotechnology, Santa Cruz, CA, USA) and anti-LTBP1 (1:500, ab78294, Abcam Inc., Cambridge, MA, USA). Sections were counterstained with haematoxylin. Sections were dehydrated with gradient alcohol solutions and 100% xylene in the reverse order as applied in the deparaffinization step, and mounted with non-aqueous media before observation under the microscope. Sections were observed by bright field microscopy (Nikon H600L, Japan). Semi-quantitative assessment was performed. The color change from light yellow to brown particles was considered as positive staining, positive cell counts were performed on 5 randomly selected fields of each slice. The scoring criteria were: i) the percent positive cells: <5% (0), 5~25% (1), 26%~50% (2), 5l% ~75% (3), >75% (4); ii) the staining intensity: light yellow (1), yellow (2), and brown (3). A final score was achieved by multiplication of the two scores, producing a total rage of 0–12. Final scores of 0, 1–4, 6–8, and 9–12 corresponded to negative, weak, moderate, and strong staining intensities, respectively.

## Results

### DPC-secreted proteins induce HF regeneration

**Fig 1:**
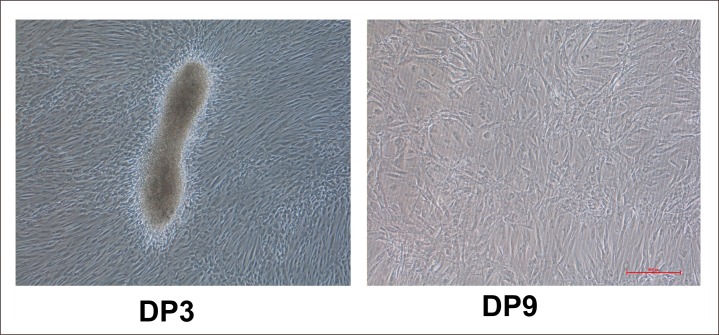
Growth conditions and morphology of DPCs. Passage 3 DPCs show aggregative behavior that is lost in passage 9 DPCs.

**Fig 2:**
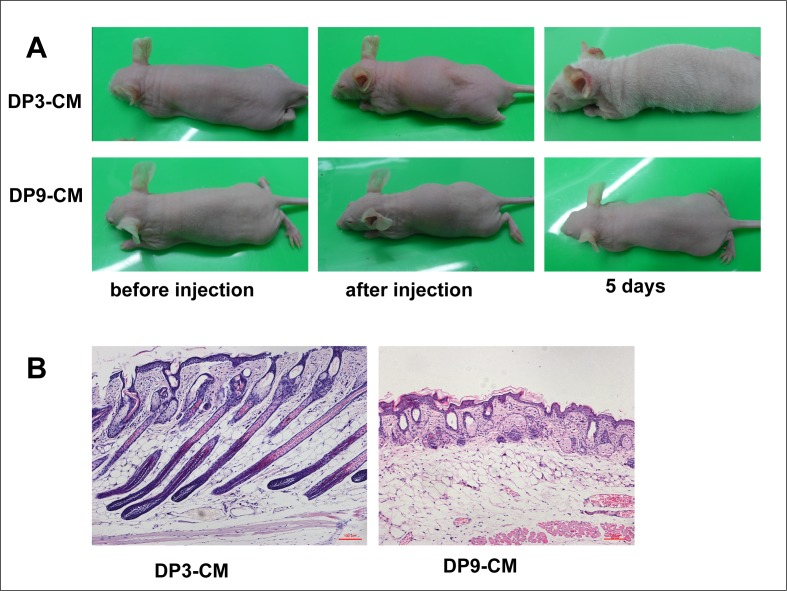
In vivo hair follicle induction by different passage DPC culture media. **(A)**Passage 3, but not passage 9 DPC-CM, induces hair generation on NU/NU mouse backs after 5 days. (**B)** Skin histology reveals HF structures in mice injected with passage 3 DPC-CM compared with mice injected with passage 9 DPC-CM.

As stated above, only early-passage DPCs show aggregative behavior. Passage 3 DP cells resembled fibroblasts and generally exhibited a multilayer aggregative behavior in culture, forming small clumps of overlapping cells (Fig 1). However, passage 9 DPCs greatly differed from passage 3 DPCs and were large and plump, and had lost aggregative behavior and proliferative capacity. In order to determine whether DPC-secreted proteins induce HF regeneration, we collected passage 3 and 9 DPC-CM, concentrated the CM through an Amicon filter, and subcutaneously injected the concentrated (2 mg/ml) CM into NU/NU mice. For passage 3 DPC-CM, a white hair coat emerged about 3 days after injection, and by 5 days, the hair fibers reached 1 cm in length, followed by hair loss after an additional 3 or 4 days. In contrast, backs of control mice, injected with passage 9 DPC-CM, remained hairless. (Fig 2A). Skin histology revealed hair follicle structures in passage 3 DPC-CM-injected mice (Fig 2B).

### Protein identification and quantitation in DPC-CM

In order to determine the difference between early- and late-passage DPC-secreted proteins, we collected passage 3 and 9 DPC-CM, after culturing for 48 h, and performed proteomic analysis. Using an iTRAQ quantitative approach, we identified a total of 1360 proteins in the culture supernatants (FDR <1%). All proteins were identified as comprising at least one peptide (>95% confidence). The 1360 proteins identified by iTRAQ are listed in S2 Table, and the identified and quantified individual peptides are listed in S3 Table. For protein quantitation, we required that a protein contain two unique spectra. The quantitative protein ratios were weighted and normalized by the median ratio in Mascot. We only considered as significant protein ratios with *p*-values <0.05, and fold changes of >1.2. There were 213 proteins that were considered significantly different in terms of expression level (*P* <0.05), including 125 proteins that were decreased (S4 Table) and 88 proteins that were increased (S5 Table) in passage 3 DPC-CM.

**Fig 3:**
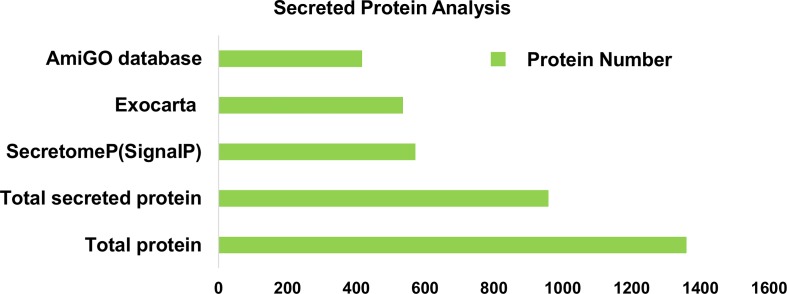
Secreted proteins identified by bioinformatic tools. Secreted proteins identified by SecretomeP, SignalP, ExoCarta and AmiGO databases.

For the subsequent analyses, we used bioinformatic tools to select, with a high level of confidence, for proteins that had been identified as secretory products. Of the 1360 proteins detected by our analysis, 959 (71%) were recognized as being secreted proteins by at least of one of the 4 bioinformatic methods. ExoCarta screening identified 536 exosome proteins, while SignalP and SecretomeP identified 572 proteins that either exhibited a signal peptide at the N-terminus or were identified as non-classical secreted products. Additionally 417 proteins were recognized as extracellular by using AmiGO, a web-based tool for searching and browsing the GO database (Fig 3). The identified secreted proteins are listed in S6 Table.

### Functional classification of the differentially-expressed proteins between early- and late-passage DPC-CM

**Fig 4:**
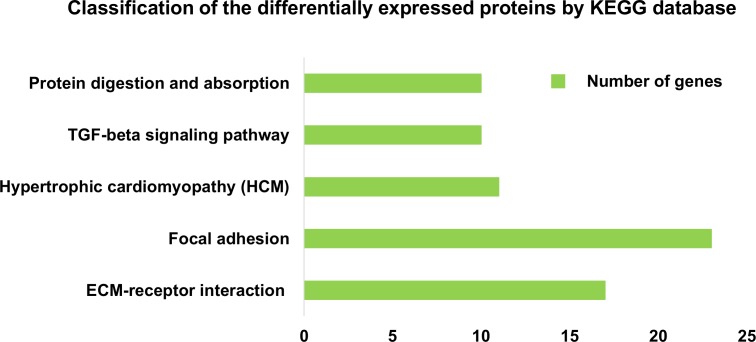
Definition of differentially expressed proteins based on KEGG analysis. Classification of secreted proteins differentially expressed between passage 3 DPC-CM and passage 9 DPC-CM, according to the KEGG database (*P*<0.01).

We used an unbiased proteomics technique to identify the critical proteins, in the DPC-CM, that regulate hair regeneration. In total, 1360 iTRAQ-labeled proteins were identified with >95% confidence. Many of these proteins were extracellular proteins, the entire list of identified extracellular proteins, as well as some important GO information and each protein are shown in S7 Table. The differentially expressed secreted proteins were further defined based on KEGG analysis (http://www.genome.jp/kegg). The proteins were mapped to KEGG pathways based on their KEGG gene ID. Proteins differentially expressed in early- and late-passage DPCs were involved in 153 KEGG pathways (S8 Table). We used a hypergeometric distribution in the enrichment analysis to prioritize these pathways. The pathways most enriched (P<0.01) involved ECM-receptor interaction, focal adhesion, hypertrophic cardiomyopathy (HCM), the TGF-β signaling pathway, protein digestion and absorption (Fig 4).

**Fig 5:**
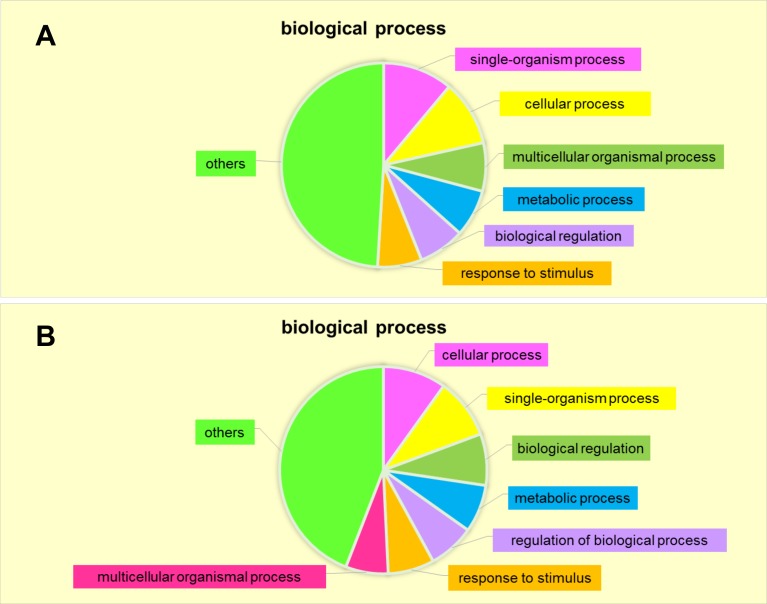
Biological processes enriched based on DPC-secreted proteins. **(A)** Up-regulated in passage 3 DPC-CM. (**B)** Down-regulated in passage 3 DPC-CM.

To gain a systematic view of secreted protein modulation during early- and late-passage DPC-CM, we analyzed the involvement of specific biological processes in the secretome responses. The proteins most highly secreted in early-passage DPCs were associated with biological processes including single-organism processes (11.07%), cellular processes (10.42%), multicellular organismal processes (7.65%), metabolic processes (7.49%), biological regulation (7.33%) and response to stimuli (7.0%) (Fig 5A). The secretome proteins most substantially down-regulated in early-passage DPC were instead associated with cellular processes (9.9%), single-organism processes (9.43%), biological regulation (8.03%), metabolic processes (7.45%), regulation of biological processes (7.22%), response to stimuli (7.22%) and multicellular organismal processes (6.64%)(Fig 5B). Thus, most of the up- and down-regulated proteins in early-passage DPC-CM participate in the same biological processes as in late-passage DPC-CM.

### Validation of the regulated secreted proteins by MRM

Although relative quantitation by iTRAQ analysis is reasonably effective at identifying increases versus decreases in protein levels, this technique is known to underestimate the true magnitude of changes in protein concentration. In order to validate the different DPC-secreted proteins identified by iTRAQ, the identified proteins were validated by MRM. Of the 213 proteins that showed a significant difference by iTRAQ analysis, we selected 19 proteins for analysis by MRM. The detailed information from the iTRAQ analysis, including the fold changes, is supplied in S9 Table. The MRM transitions of each targeted peptide and CE (Collision Energy) is detailed in S10 Table, and the MRM ratio is detailed in S11 Table. As a result, the 19 proteins were successfully quantified, and all the target proteins showed the same trend as when quantified by iTRAQ (Table 1).

**Table 1:** iTRAQ-based quantification compared with MRM-based quantification

Gene symbol	Uniprot accession	Protein name	Unique peptides(iTRAQ)	iTRAQ ratios DP3/DP9(Mean)	MRM ratios DP3/DP9	Expression pattern
**PLOD2**	**sp|O00469**	**Procollagen-lysine,2-oxoglutarate 5-dioxygenase 2**	**7**	**0.499**	**0.438**	**down**
**DCN**	**sp|P07585**	**Decorin**	**8**	**4.603**	**12.555**	**up**
**THBS1**	**sp|P07996**	**Thrombospondin-1**	**1**	**0.272**	**0.579**	**down**
**MMP3**	**sp|P08254**	**Stromelysin-1**	**1**	**3.11**	**4.313**	**up**
**COL4A2**	**sp|P08572**	**Collagen alpha-2(IV) chain**	**16**	**0.592**	**0.285**	**down**
**ACTN1**	**sp|P12814**	**Alpha-actinin-1**	**22**	**0.564**	**0.475**	**down**
**IGFBP4**	**sp|P22692**	**Insulin-like growth factor-binding protein 4**	**4**	**1.601**	**1.755**	**up**
**FBLN1**	**sp|P23142**	**Fibulin-1**	**5**	**1.977**	**10.989**	**up**
**IGFBP5**	**sp|P24593**	**Insulin-like growth factor-binding protein 5**	**4**	**0.381**	**0.21**	**down**
**SERPINF1**	**sp|P36955**	**Pigment epithelium-derived factor**	**10**	**5.711**	**80.361**	**up**
**CXCL12**	**sp|P48061**	**Stromal cell-derived factor 1**	**2**	**4.7**	**13.229**	**up**
**ACLY**	**sp|P53396**	**ATP-citrate synthase**	**6**	**0.497**	**0.581**	**down**
**FMOD**	**sp|Q06828**	**Fibromodulin**	**2**	**4.982**	**8.867**	**up**
**FLNC**	**sp|Q14315**	**Filamin-C**	**26**	**0.488**	**0.295**	**down**
**LTBP1**	**sp|Q14766**	**Latent-transforming growth factor beta-binding protein 1**	**8**	**3.039**	**5.93**	**up**
**AEBP1**	**sp|Q8IUX7**	**Adipocyte enhancer-binding protein 1**	**12**	**3.201**	**8.103**	**up**
**SPON2**	**sp|Q9BUD6**	**Spondin-2**	**7**	**1.269**	**1.649**	**up**
**PDGFD**	**sp|Q9GZP0**	**Platelet-derived growth factor D**	**3**	**2.267**	**2.516**	**up**
**DPP7**	**sp|Q9UHL4**	**Dipeptidyl peptidase 2**	**6**	**1.876**	**2.338**	**up**

To examine the correlation of the quantitation data between MRM and iTRAQ analyses, we compared the expression level of proteins obtained by MRM with that of iTRAQ (Table 2). The correlation coefficient is calculated from the 3 repeated ratios and their mean ratio. Procollagen-lysine, stromelysin-1, insulin-like growth factor-binding protein 4, insulin-like growth factor-binding protein 5, pigment epithelium-derived factor, filamin-C, adipocyte enhancer-binding protein 1, platelet-derived growth factor D, and dipeptidyl peptidase 2 were highly correlated between iTRAQ and SRM (r^2^ > 0.6), whereas decorin, collagen alpha-2 (IV) chain, stromal cell-derived factor 1, and ATP-citrate synthase were less well correlated (r^2^> 0.4 to < 0.6), and thrombospondin-1, alpha-actinin-1, fibulin-1, fibromodulin, latent-transforming growth factor beta-binding protein 1, and spondin-2 showed no correlation. The reason for this discrepancy might be due to the low abundance of peptides, small sample size and complicated procedure of proteomic analysis without suitable internal standards (11).

**Table 2:** Linear regression comparing peptide ratio results obtained by iTRAQ and MRM assays.

Gene symbol	Uniprot accession	Protein name	Correlation coefficients, r^2^
**PLOD2**	**sp|O00469**	**Procollagen-lysine,2-oxoglutarate 5-dioxygenase 2**	**0.9973**
**MMP3**	**sp|P08254**	**Stromelysin-1**	**0.7987**
**IGFBP4**	**sp|P22692**	**Insulin-like growth factor-binding protein 4**	**0.9874**
**IGFBP5**	**sp|P24593**	**Insulin-like growth factor-binding protein 5**	**0.7582**
**SERPINF1**	**sp|P36955**	**Pigment epithelium-derived factor**	**0.9817**
**FLNC**	**sp|Q14315**	**Filamin-C**	**0.9123**
**AEBP1**	**sp|Q8IUX7**	**Adipocyte enhancer-binding protein 1**	**0.8703**
**PDGFD**	**sp|Q9GZP0**	**Platelet-derived growth factor D**	**0.8682**
**DPP7**	**sp|Q9UHL4**	**Dipeptidyl peptidase 2**	**0.8674**
**DCN**	**sp|P07585**	**Decorin**	**0.5686**
**COL4A2**	**sp|P08572**	**Collagen alpha-2(IV) chain**	**0.4556**
**CXCL12**	**sp|P48061**	**Stromal cell-derived factor 1**	**0.4819**
**ACLY**	**sp|P53396**	**ATP-citrate synthase**	**0.5589**
**THBS1**	**sp|P07996**	**Thrombospondin-1**	**0.0015**
**ACTN1**	**sp|P12814**	**Alpha-actinin-1**	**0.0303**
**FBLN1**	**sp|P23142**	**Fibulin-1**	**0.036**
**FMOD**	**sp|Q06828**	**Fibromodulin**	**0.0679**
**LTBP1**	**sp|Q14766**	**Latent-transforming growth factor beta-binding protein 1**	**0.0864**
**SPON2**	**sp|Q9BUD6**	**Spondin-2**	**0.0114**

### Expression of SDF1, MMP3, biglycan and LTBP1

**Fig 6:**
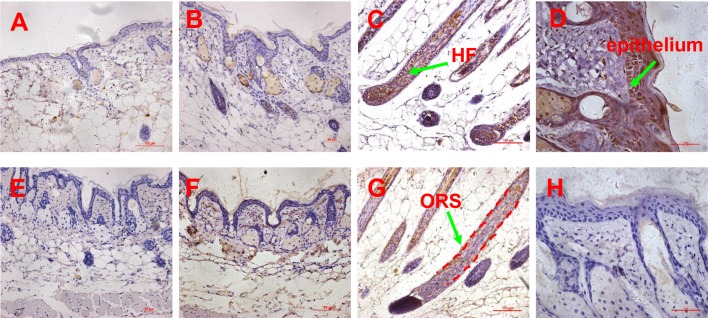
Expression pattern of SDF1 and MMP3 proteins determined by immunohistochemistry. (A-B) the expression of SDF1 in the skin of untreated and passage 9 DPC-CM injected mice (20x objective), (C) the expression of SDF1 in the skin of passage 3 DPC-CM injected mice (20x objective), (D) the expression of SDF1 in the epithelium of skin form passage 3 DPC-CM injected mice (40x objective); (E-F) the expression of MMP3 in the skin of untreated and passage 9 DPC-CM injected mice (20x objective), (G) the expression of MMP3 in the ORS of skin from passage 3 DPC-CM injected mice (20x objective), (H) the expression of MMP3 in the epithelium of passage 3 DPC-CM injected mice skin (40x objective).

**Fig 7:**
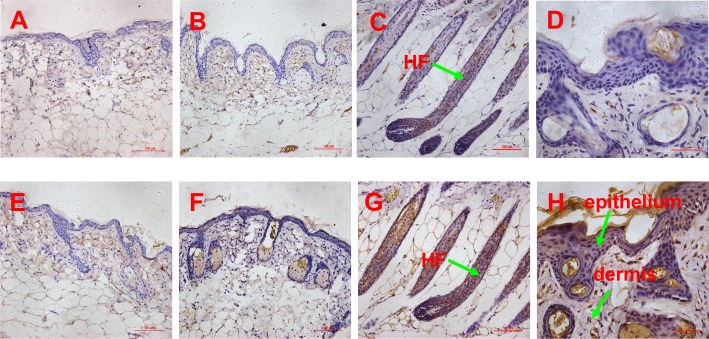
Expression pattern of biglycan and LTBP1 proteins determined by immunohistochemistry. (A-B) the expression of biglycan in the skin of untreated and passage 9 DPC-CM injected mice (20x objective), (C) the expression of biglycan in the skin of passage 3 DPC-CM injected mice (20x objective), (D) the expression of biglycan in the epithelium of skin form passage 3 DPC-CM injected mice (40x objective); (E-F) the expression of LTBP1 in the skin of untreated and passage 9 DPC-CM injected mice (20x objective), (G) the expression of LTBP1 in the ORS of skin from passage 3 DPC-CM injected mice (20x objective), (H) the expression of LTBP1 in the epithelium of skin from passage 3 DPC-CM injected mice (40x objective).

In order to confirm SDF1, MMP3 and LTBP1 to be potential key factors in HF regeneration, tissues of passage 3 DPC-CM-injected, passage 9 DPC-CM injected and untreated mice were obtained to determine their expression patterns by immunohistochemistry. Also, the expression pattern of biglycan known to play key roles in many cellular signaling pathways involved in hair follicle biology was also detected. In the skin of passage 9 DPC-CM injected mice and untreated mice, SDF1 (Fig 6A and 6B), MMP3 (Fig 6E and 6F), biglycan (Fig 7A and 7B) and LTBP1 (Fig 7E and 7F) were rarely expressed. Interestingly, SDF1 (Fig 6C) and biglycan (Fig 7C), as well as LTBP1 (Fig 7G) proteins were all expressed in hair follicles, with the highest expression of SDF1 (Fig 6D) being located to the epithelial cells in the skin from passage 3 DPC-CM-injected mice. In addition, MMP3 was expressed only in the ORS of passage 3 DPC-CM-injected mice skin (Fig 6G), whereas LTBP1 was strongly expressed in epithelial tissue and dermal cells (Fig 7H).

## Discussion

DPCs secrete diverse growth factors and stimulate the proliferation and differentiation of the follicular epithelium, implying DPC-secreted paracrine factors regulate HF regeneration [19]. We show that passage 3 DPC-CM induces HF regeneration, but passage 9 DPC-CM does not. These results indicate DPC-secreted proteins play important roles in HF regeneration. Analysis of CM from DPCs, by iTRAQ-based quantitative proteomic technology, identified 1360 proteins, of which 213 proteins were differentially expressed between CM from early-passage vs. late- passage DPCs. KEGG enrichment analysis showed that the differentially expressed proteins are involved in 153 KEGG pathways. Among the most enriched pathways (P<0.01), the TGF-β signaling pathway has been shown to be essential for regeneration, as it activates the Smad2/3 pathway in HF stem cells, which is crucial for avoiding delayed regeneration [3].

For the subsequent analyses, we used bioinformatic tools (ExoCarta, SignalP, SecretomeP, AmiGO) to select proteins previously identified as secretory products [20]. Of the 1360 proteins detected by our analysis, 959 (71%) have been previously recognized, as being secreted, by one of the 4 bioinformatic methods. These results support the reliability of our data. In order to validate the different DPC-secreted proteins identified by iTRAQ, we selected 12 overexpressed proteins and 7 underexpressed polypeptides, from early-passage DPC-CM, for validation, of differential protein expression, by MRM, and to verify key candidate proteins regulating HF regeneration. The validated proteins were selected according to their GO function annotation and involvement in signal pathways that are relevant to hair follicle (HF) regeneration, such as the Wnt, BMP, TGF-β, and IGF1 pathways [3]. Successful validation provides a high confidence level for the secreted proteins identified by iTRAQ.

In this study, we focus on identifying the key factors responsible for hair inductive capacity. HF regeneration involves BMP antagonism and subsequent activation of Wnt and other underlying pathways. The initial steps of regeneration involve crosstalk between quiescent epithelial stem cells and mesenchymal dermal papilla, forming a repressive environment for BMP signaling [3, 21]. In our proteomic analysis, we searched for factors involved in the Wnt and BMP pathways and identified SDF1, MMP3 and biglycan, which participate in the Wnt signaling pathway, as well as LTBP1, which participates in the BMP signaling pathway.

We identified stromal cell-derived factor 1 (SDF1, also known as CXCL12) as being one of the most overexpressed in early-passage DPCs, compared to late passage DPCs. SDF-1 is a ligand for CXCR4, is a powerful chemoattractant for stem cells, and plays an important role in several developmental and regenerative phenomena, such as cardiogenesis, neovascularization, hematopoiesis, and hepatic development [22]. SDF-1 is localized to the hair follicle dermal papilla (DP), but is excluded from the epidermis. CXCR4 is specifically expressed in the hair follicle dermal papilla, where it is localized to a few epidermal cells at the mouth of developing hair follicles. The close juxtaposition and complementary nature of CXCR4 and SDF-1 expression patterns suggest that SDF-1/CXCR4 signaling might play a role in HF development [23]. However, in our induced HF regeneration, compared with passage 9 DPC-CM injected mice, SDF1 was strongly expressed not only in the HF, but also in the epithelium of passage 3 DPC-CM-injected mice. Those findings indicate that SDF1 may be a key factor for initiating hair follicle regeneration. A recent report suggests that SDF-1 promotes epidermal stem cell (ESC) migration and accelerates wound healing in vivo [22]. Based on previous studies, SDF1 has the potential to regulate HF regeneration. However, the molecular mechanisms have not been demonstrated. Based on the interaction between SDF1/CXCR4 signaling and Wnt/β-catenin in some cancer cells and neural progenitors [24–26] and the important role in HF initiation as well as primary hair placode maintenance of Wnt/β-catenin signaling pathway, we hypothesize that SDF1 may participate in HF regeneration by regulating Wnt/β-catenin signaling.

**Fig 8:**
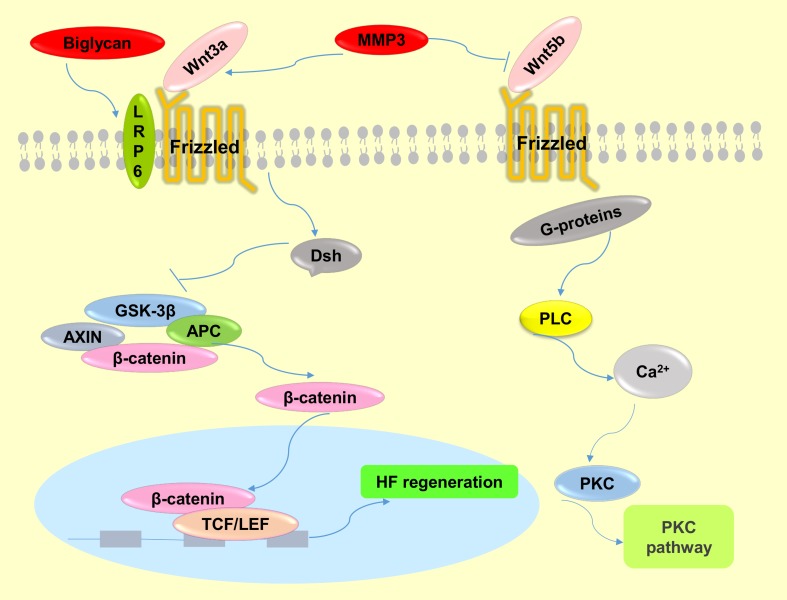
The molecular mechanisms of Wnt signaling pathway regulation by MMP3 and biglycan. MMP3 activates the canonical Wnt signaling pathway by cooperating with Wnt3a or antagonizing Wnt5b function, which is a non-canonical Wnt ligand and inhibitor of Wnt/β-catenin signaling. Biglycan enhances Wnt-induced β-catenin/TCF-mediated transcriptional activity via the LRP6 receptor.

Another identified DPC-secreted factor, matrix metallopeptidase 3 (MMP3), belongs to the matrix metalloproteinase (MMP) family of proteases that has been implicated in a large variety of physiological and pathological conditions associated with tissue remodeling [27]. Although the function of MMP3 in HF regeneration has not yet been reported, MMP can modify the microenvironment of stem cells in bone, and MMP3 has been shown to have an impact on the maintenance of adult epithelial stem cells [28], implying MMP3, which we show is increased in early passage DPCs, has the potency to modify the hair follicle stem cell (HFSC) microenvironment “niche”, which plays a crucial role in cell fate decision. MMP3 could affect adult epithelial stem cells though activating Wnt/β-catenin signaling by antagonizing the function of Wnt5b (Fig 8), a non-canonical Wnt ligand and inhibitor of Wnt/β-catenin signaling [29]. Another report showed that MMP3 cooperates with Wnt3a to increase the transcriptional activity of β–catenin in C57MG cells (Fig 8) [27]. Additionally, a recent study suggested that MMPs serve as paracrine factors to activate Wnt signaling in mesenchymal stem cells (MSCs) [30]. It is well known that the Wnt signaling pathway plays a crucial role in the maintenance and proliferation of HFSC reservoirs [3]. In addition, MMP3 was highly expressed in the ORS of passage 3 DPC-CM-injected mice, which is close to the bulge where hair follicle stem cells (HFSCs) are located. Taken together, we speculate that MMP3 participates in the process of HF regeneration by activating Wnt/β–catenin in HFSCs (Fig 8).

A third important factor is biglycan, a member of the small leucine-rich proteoglycan family, and an important structural component of the ECM [31, 32]. Biglycan functions as a signaling molecule released from the ECM. Proteoglycans are known to play key roles in many cellular signaling pathways involved in hair follicle biology [33]. Interestingly, although the expression level of biglycan was increased in the skin of passage 3 DPC-CM-injected mice and was highly expressed in the induction of HFs, it was rarely expressed in the skin of passage 9 DPC-CM injected mice in this study, consistent with a prior demonstration that the DP contains a high level of biglycan [33], and suggests that biglycan is involved in key functions of HF biology. Furthermore, both biglycan proteoglycan and biglycan core protein enhance Wnt-induced β-catenin/TCF-mediated transcriptional activity via the LRP6 receptor (Fig 8) [31]. Recently, additional research has demonstrated that biglycan, as a component of the ECM, mediates suture expansion osteogenesis through the Wnt/β-catenin signaling-activated Runx2 pathway [32]. Therefore, it is possible that DPC secretion of biglycan impacts HF regeneration by also regulating the Wnt/β-catenin signaling pathway (Fig 8).

Although, we do not discuss the direct regulators involved in BMP antagonists in our study, we confirmed that latent TGF-β-binding protein 1 (LTBP1) is over-expressed in passage 3 DPC-CM. In addition, we show that LTBP1 was not expressed in the skin of passage 9 DPC-CM injected mice, but was strongly expressed in the regenerated HF, epithelium and dermis. LTBP-1 covalently binds to the small latent TGF-β complex and regulates its function, presumably via interaction with the ECM (34). Several prior reports illustrate that LTBP1 is a positive modulator of TGF-β1 activation [34–36], and is considered an antagonist of BMP4 signaling through activating TGF-β1 signaling via the TGF-β1 type I receptor [37]. Dermal papillae produce a paracrine TGF-β2 signal to activate canonical TGF-β-mediated transcription and induce proliferation in quiescent HFSCs, and propels them into a tissue-regenerating mode. Additionally, BMP signaling is prolonged when HFSCs cannot respond to TGF-β2. Tmeff1, a relevant TGF-β2 target gene, is an antagonist of the BMP pathway. Intriguingly, LTBP1 increases TGF-β1 and TGF-β2 at the same time in glioma cells [34].Thus, LTBP1 could inhibit BMP signaling by activating the TGF-β signaling pathway.

## Conclusion

Our results suggest that DPCs regulate hair follicle regeneration in paracrine fashion by secreting SDF1, MMP3 and biglycan, to activate Wnt/β-catenin signaling, and LTBP1 to block BMP signaling, as part of establishing a microenvironment for the induction of HF regeneration.

## Supporting Information

S1 Table Selected peptides for 19 target proteins. Details of all the peptides used in this study and their corresponding proteins were listed.
(XLS)

S2 Table Proteins identified by iTRAQ. There were 1360 proteins identified from CM of early-passage and late-passage DPCs by iTRAQ.
(XLS)

S3 Table Identified and quantified individual peptides. All the identified and quantified individual peptides were listed.
(XLS)

S4 Table Decreased proteins in passage 3 DPC-CM. There were 125 proteins decreasing in passage 3 DPC-CM.
(XLS)

S5 Table Increased proteins in passage 3 DPC-CM. There were 88 proteins increasing in passage 3 DPC-CM.
(XLS)

S6 Table Secreted proteins identified by bioinformatics tools. Of the 1360 proteins, 959 (71%) were recognized as being secreted proteins by at least of one of the 4 bioinformatic methods.
(XLS)

S7 Table GO information of each identified protein. The entire list of identified extracellular proteins and some important GO information were shown.
(XLS)

S8 Table KEGG analysis. Proteins differentially expressed between CM from early- vs. late-passage DPCs were involved in 153 KEGG pathways.
(XLS)

S9 Table Detailed information from iTRAQ analysis of the 19 target proteins. Detailed information from the iTRAQ analysis of the 19 target proteins, including the fold changes was displayed.
(XLS)

S10 Table Optimization of MRM transitions. It showed the MRM transitions of each targeted peptide and CE.
(XLS)

S11 Table MRM ratios. The detailed MRM ratios were listed.
(XLS)

## Abbreviations

DPCs: dermal papilla cells
HF: hair follicle
CM: culture medium
DPC-CM: dermal papilla cell culture medium
DP: dermal papilla
MRM: multiple reaction monitoring
ECM: extracellular matrix
iTRAQ: isobaric tag for relative and absolute quantitation
APA: alginate-polylysine-alginate
Shh: Sonic Hedgehog
BMP: Bone morphogenetic protein
DMEM: Dulbecco´s modified Eagle’s medium
FBS: fetal bovine serum
TEAB: triethylammonium bicarbonate
BCA: bicinchoninic acid
RP: Reverse Phase
IDA: Information Dependant Acquistion
HCM: hypertrophic cardiomyopathy
CE: Collision Energy
DS: dermal matrix
IRS: inner root sheath
ORS: outer root sheath
ESC: epidermal stem cell
SDF1: stromal cell-derived factor 1
MMP3: matrix metallopeptidase 3
MMP: matrix metalloproteinase
MSCs: mesenchymal stem cells
HFSC: hair follicle stem cells

## References

[1] Balana ME, Charreau HE, Leiros GJ. Epidermal stem cells and skin tissue engineering in hair follicle regeneration. World journal of stem cells. 2015;7(4):711–27. 10.4252/wjsc.v7.i4.711 26029343 PMC4444612

[2] Zhang P, Kling RE, Ravuri SK, Kokai LE, Rubin JP, Chai JK, et al A review of adipocyte lineage cells and dermal papilla cells in hair follicle regeneration. Journal of tissue engineering. 2014;5:2041731414556850. 10.1177/2041731414556850 PMC4221925 25383178

[3] Rishikaysh P, Dev K, Diaz D, Qureshi WM, Filip S, Mokry J. Signaling involved in hair follicle morphogenesis and development. International journal of molecular sciences. 2014;15(1):1647–70. 10.3390/ijms15011647 24451143 PMC3907891

[4] Inamatsu M, Matsuzaki T, Iwanari H, Yoshizato K. Establishment of rat dermal papilla cell lines that sustain the potency to induce hair follicles from afollicular skin. The Journal of investigative dermatology. 1998;111(5):767–75. 10.1046/j.1523-1747.1998.00382.x 9804336

[5] Won CH, Kwon OS, Kang YJ, Yoo HG, Lee DH, Chung JH, et al Comparative secretome analysis of human follicular dermal papilla cells and fibroblasts using shotgun proteomics. BMB reports. 2012;45(4):253–8. 22531137 10.5483/bmbrep.2012.45.4.253

[6] Fujie T, Katoh S, Oura H, Urano Y, Arase S. The chemotactic effect of a dermal papilla cell-derived factor on outer root sheath cells. Journal of dermatological science. 2001;25(3):206–12. 11240268 10.1016/s0923-1811(00)00130-4

[7] Hamada K, Randall VA. Inhibitory autocrine factors produced by the mesenchyme-derived hair follicle dermal papilla may be a key to male pattern baldness. The British journal of dermatology. 2006;154(4):609–18. 10.1111/j.1365-2133.2006.07144.x 16536801

[8] Lin CM, Li Y, Ji YC, Keng H, Cai XN, Zhang JK. Microencapsulated human hair dermal papilla cells: a substitute for dermal papilla? Archives of dermatological research. 2008;300(9):531–5. 10.1007/s00403-008-0852-3 18418617

[9] Lin CM, Li Y, Ji YC, Huang K, Cai XN, Li GQ. Induction of hair follicle regeneration in rat ear by microencapsulated human hair dermal papilla cells. Chinese journal of traumatology = Zhonghua chuang shang za zhi / Chinese Medical Association. 2009;12(1):49–54. 19159517

[10] Zhang F, Lin H, Gu A, Li J, Liu L, Yu T, et al SWATH- and iTRAQ-based quantitative proteomic analyses reveal an overexpression and biological relevance of CD109 in advanced NSCLC. Journal of proteomics. 2014;102:125–36. 10.1016/j.jprot.2014.03.007 24667143

[11] Narumi R, Murakami T, Kuga T, Adachi J, Shiromizu T, Muraoka S, et al A strategy for large-scale phosphoproteomics and SRM-based validation of human breast cancer tissue samples. Journal of proteome 7research. 2012;11(11):5311–22. 10.1021/pr3005474 22985185

[12] Li Y, Li GQ, Lin CM, Cai XN. One-step collagenase I treatment: an efficient way for isolation and cultivation of human scalp dermal papilla cells. Journal of dermatological science. 2005;37(1):58–60. 10.1016/j.jdermsci.2004.10.001 15619437

[13] Karagiannis GS, Pavlou MP, Diamandis EP. Cancer secretomics reveal pathophysiological pathways in cancer molecular oncology. Molecular oncology. 2010;4(6):496–510. 10.1016/j.molonc.2010.09.001 20934395 PMC5527923

[14] Bendtsen JD, Nielsen H, von Heijne G, Brunak S. Improved prediction of signal peptides: SignalP 3.0. Journal of molecular biology. 2004;340(4):783–95. 10.1016/j.jmb.2004.05.028 15223320

[15] Bendtsen JD, Jensen LJ, Blom N, Von Heijne G, Brunak S. Feature-based prediction of non-classical and leaderless protein secretion. Protein engineering, design & selection: PEDS. 2004;17(4):349–56. 10.1093/protein/gzh037 15115854

[16] Yan H, Zhou W, Wei L, Zhong F, Yang Y. Proteomic analysis of astrocytic secretion that regulates neurogenesis using quantitative amine-specific isobaric tagging. Biochemical and biophysical research communications. 2010;391(2):1187–91. 10.1016/j.bbrc.2009.12.015 20005204

[17] Mathivanan S, Simpson RJ. ExoCarta: A compendium of exosomal proteins and RNA. Proteomics. 2009;9(21):4997–5000. 10.1002/pmic.200900351 19810033

[18] MacLean B, Tomazela DM, Shulman N, Chambers M, Finney GL, Frewen B, et al Skyline: an open source document editor for creating and analyzing targeted proteomics experiments. Bioinformatics (Oxford, England). 2010;26(7):966–8. 10.1093/bioinformatics/btq054 PMC2844992 20147306

[19] Yang CC, Cotsarelis G. Review of hair follicle dermal cells. Journal of dermatological science. 2010;57(1):2–11. 10.1016/j.jdermsci.2009.11.005 20022473 PMC2818774

[20] Li X, Ren Y, Sorokin V, Poh KK, Ho HH, Lee CN, et al Quantitative profiling of the rat heart myoblast secretome reveals differential responses to hypoxia and re-oxygenation stress. Journal of proteomics. 2014;98:138–49. 10.1016/j.jprot.2013.12.025 24412200

[21] Plikus MV, Chuong CM. Macroenvironmental regulation of hair cycling and collective regenerative behavior. Cold Spring Harbor perspectives in medicine. 2014;4(1):a015198 10.1101/cshperspect.a015198 24384813 PMC3869280

[22] Guo R, Chai L, Chen L, Chen W, Ge L, Li X, et al Stromal cell-derived factor 1 (SDF-1) accelerated skin wound healing by promoting the migration and proliferation of epidermal stem cells. In vitro cellular & developmental biology Animal. 2015;51(6):578–85. 25636237 10.1007/s11626-014-9862-y

[23] Belmadani A, Jung H, Ren D, Miller RJ. The chemokine SDF-1/CXCL12 regulates the migration of melanocyte progenitors in mouse hair follicles. Differentiation; research in biological diversity. 2009;77(4):395–411. 10.1016/j.diff.2008.10.015 19281787 PMC4461245

[24] Hu TH, Yao Y, Yu S, Han LL, Wang WJ, Guo H, et al SDF-1/CXCR4 promotes epithelial-mesenchymal transition and progression of colorectal cancer by activation of the Wnt/beta-catenin signaling pathway. Cancer letters. 2014;354(2):417–26. 10.1016/j.canlet.2014.08.012 25150783

[25] Wang Z, Ma Q, Liu Q, Yu H, Zhao L, Shen S, et al Blockade of SDF-1/CXCR4 signalling inhibits pancreatic cancer progression in vitro via inactivation of canonical Wnt pathway. British journal of cancer. 2008;99(10):1695–703. 10.1038/sj.bjc.6604745 19002187 PMC2584946

[26] Luo Y, Cai J, Xue H, Mattson MP, Rao MS. SDF1alpha/CXCR4 signaling stimulates beta-catenin transcriptional activity in rat neural progenitors. Neuroscience letters. 2006;398(3):291–5. 10.1016/j.neulet.2006.01.024 16469439

[27] Blavier L, Lazaryev A, Shi XH, Dorey FJ, Shackleford GM, DeClerck YA. Stromelysin-1 (MMP-3) is a target and a regulator of Wnt1-induced epithelial-mesenchymal transition (EMT). Cancer biology & therapy. 2010;10(2):198–208. 20534975 10.4161/cbt.10.2.12193 PMC3040898

[28] Kessenbrock K, Wang CY, Werb Z. Matrix metalloproteinases in stem cell regulation and cancer. Matrix biology: journal of the International Society for Matrix Biology. 2015;44–46:184–90. 10.1016/j.matbio.2015.01.022 PMC4498798 25661772

[29] Kessenbrock K, Dijkgraaf GJ, Lawson DA, Littlepage LE, Shahi P, Pieper U, et al A novel role for matrix metalloproteinases in regulating mammary stem cell function via the Wnt signaling pathway. Cell stem cell. 2013;13(3):300–13. 10.1016/j.stem.2013.06.005 23871604 PMC3769456

[30] Chen D, Liu S, Ma H, Liang X, Ma H, Yan X, et al Paracrine factors from adipose-mesenchymal stem cells enhance metastatic capacity through Wnt signaling pathway in a colon cancer cell co-culture model. Cancer cell international. 2015;15:42 10.1186/s12935-015-0198-9 26060426 PMC4460851

[31] Berendsen AD, Fisher LW, Kilts TM, Owens RT, Robey PG, Gutkind JS, et al Modulation of canonical Wnt signaling by the extracellular matrix component biglycan. Proceedings of the National Academy of Sciences of the United States of America. 2011;108(41):17022–7. 10.1073/pnas.1110629108 21969569 PMC3193219

[32] Wang H, Sun W, Ma J, Pan Y, Wang L, Zhang WB. Biglycan mediates suture expansion osteogenesis via potentiation of Wnt/beta-catenin signaling. Journal of biomechanics. 2015;48(3):432–40. 10.1016/j.jbiomech.2014.12.032 25560274

[33] Malgouries S, Thibaut S, Bernard BA. Proteoglycan expression patterns in human hair follicle. The British journal of dermatology. 2008;158(2):234–42. 10.1111/j.1365-2133.2007.08339.x 18067481

[34] Tritschler I, Gramatzki D, Capper D, Mittelbronn M, Meyermann R, Saharinen J, et al Modulation of TGF-beta activity by latent TGF-beta-binding protein 1 in human malignant glioma cells. International journal of cancer Journal international du cancer. 2009;125(3):530–40. 10.1002/ijc.24443 19431147

[35] Gomez-Duran A, Mulero-Navarro S, Chang X, Fernandez-Salguero PM. LTBP-1 blockade in dioxin receptor-null mouse embryo fibroblasts decreases TGF-beta activity: Role of extracellular proteases plasmin and elastase. Journal of cellular biochemistry. 2006;97(2):380–92. 10.1002/jcb.20637 16187295

[36] Todorovic V, Frendewey D, Gutstein DE, Chen Y, Freyer L, Finnegan E, et al Long form of latent TGF-beta binding protein 1 (Ltbp1L) is essential for cardiac outflow tract septation and remodeling. Development (Cambridge, England). 2007;134(20):3723–32. 10.1242/dev.008599 17804598

[37] Upton PD, Davies RJ, Tajsic T, Morrell NW. Transforming growth factor-beta(1) represses bone morphogenetic protein-mediated Smad signaling in pulmonary artery smooth muscle cells via Smad3. American journal of respiratory cell and molecular biology. 2013;49(6):1135–45. 10.1165/rcmb.2012-0470OC 23937428 PMC3931109

